# Management of Bilateral Congenital Lacrimal Punctal and Canalicular Atresia and Congenital Fistula of the Lacrimal Sac

**DOI:** 10.4103/0974-9233.63075

**Published:** 2010

**Authors:** Shreya Shah, Mehul Shah, Rajiv Khandekar

**Affiliations:** Drashti Netralaya, Dahod, Gujarat, India

**Keywords:** Canalicular Atresia, Congenital Fistula, Congenital Punctal

## Abstract

An 8-year-old girl presented with complaints of bilateral epiphora since birth. The patient had congenital punctal and canalicular atresia combined with congenital fistula. She was treated successfully with surgery. A review of the literature indicated very few reports of surgical treatment of such cases.

## INTRODUCTION

Morphogenesis of the lacrimal system begins at week 6 of gestation. Many congenital anomalies of the lacrimal system may occur if development is affected during this time.^1^,^2^ Common anomalies are dacryostenosis, sac diverticula, punctal atresia, displacement, canalicular atresia, failure of canalization, and congenital fistul^1^–^3^ These anomalies can cause partial or total obstruction of the lacrimal drainage system.

## CASE REPORT

**Figure 1:**
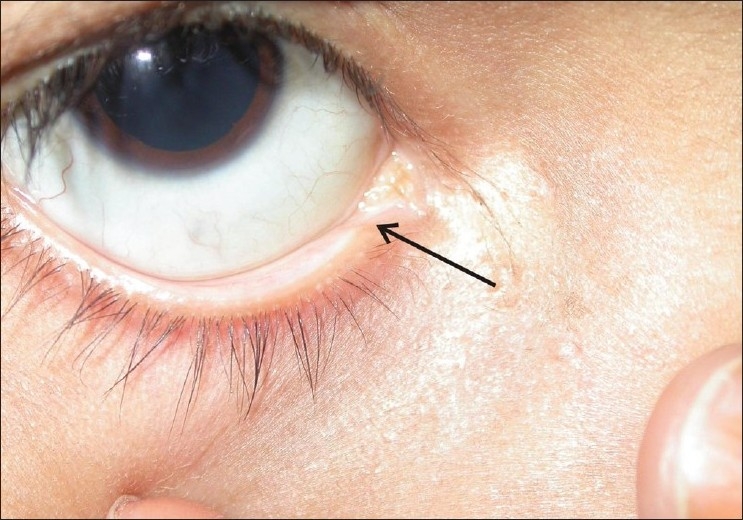
Absence of punctum

**Figure 2:**
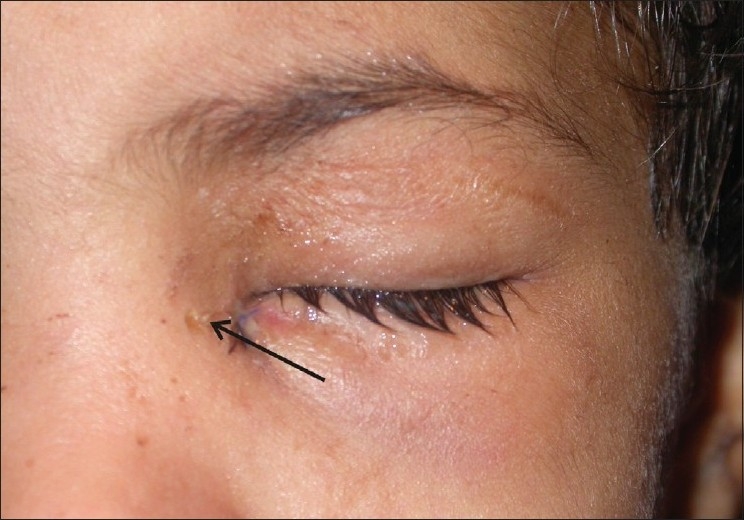
View of cutaneous fistula

An 8-year-old girl presented with a chief complaint of watery discharge bilaterally since birth. Slit lamp examination of the anterior segment and indirect ophthalmoscopy of the posterior segment were unremarkable. Motor development was normal. The absence of puncta and canaliculi was noted bilaterally on examination of the lacrimal system. An opening was present 6–7 mm from inner canthus on the cheek. Syringing of the cutaneous opening of this cheek fistula revealed communication with the nose [Figures 1 and 2]. The presence of atresia of puncta and canaliculi bilaterally was confirmed.

**Figure 3:**
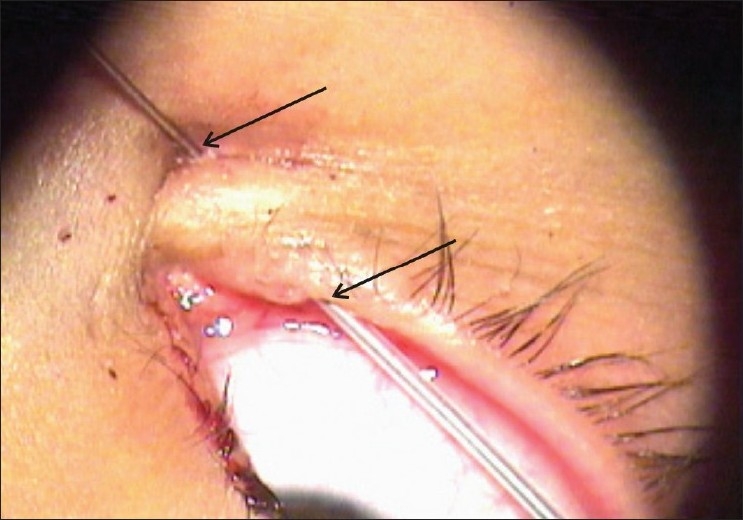
Probe reaching the nasal opening

**Figure 4:**
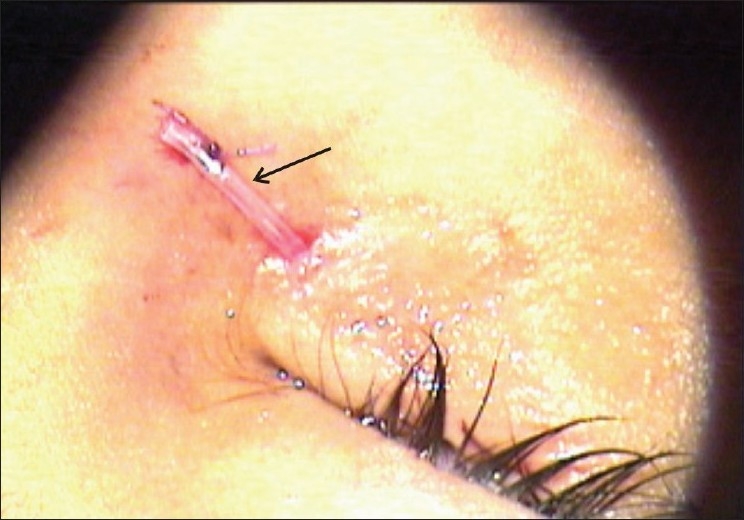
Teflon tube inserted from the proposed punctal site leading to the fistula opening

Surgical treatment was performed under general anesthesia. The site of the puncta was marked in both eyes. A lacrimal probe was passed from the fistulous opening into the lacrimal sac with a 23-gauge intracath with insertion of a teflon sleeve at the site of the lower punctum which was directed downward and slightly posterior once it contacted the probe [Figures 3 and 4]. The trocar was removed, and fluid in the sleeve was expressed freely at the fistulous opening. The probe was inserted through the skin opening into the nose in the direction of the nasolacrimal duct and a tube was directed toward the nasolacrimal duct to confirm the patency. The passage of fluid from the nose to the throat was confirmed by the presence of blue stain on endoscopy. A teflon tube was fixed at the lid margin with an 8-0 vicryl suture. A fistulectomy was performed and the opening closed with 6-0 catgut, and the skin was sutured with 6-0 Vicryl.

The child was examined periodically to ensure the patency of the lacrimal drainage system of both eyes. On last examination at 3 years postoperatively, both lacrimal passages were functional. The patency was tested with lacrimal sac syringing and fluoresceine dye. Radio-imaging with dye confirmed the patency of the lacrimal drainage system bilaterally. The child claimed resolution of epiphora postoperatively.

## DISCUSSION

The presence of congenital punctal, canalicular atresia, and congenital fistula are a extremely rare combination.^1^,^2^ Documentation of surgical treatment of this combined bilateral anomaly is also very rare^3^. In this case, management options were limited as no punctae were visible.

Congenital lacrimal fistulas are routinely managed with fistulectomy and dacryocyctorhinostomy.^4^,^5^ The case reported here had atresia of the lacrimal drainage system along with congenital fistula. Bilateral congenital lacrimal anlagen ducts (lacrimal fistula) in a patient with the VACTERL association (vertebral anomalies, anal atresia, cardiac malformations, tracheo-esophageal fistula, renal anomalies, and limb anomalies) has been previously reported.^5^ However, the child in the current report, had no systemic or congenital anomalies. Treatment of combined congenital anomalies of the lacrimal system, as managed in this study is feasible. Cahill and Burns have reported that combination of anomalies like punctal and canalicular atresia, with congenital lacrimal fistula, can be treated successfully by surgical correction.^6^ Our experience with the case presented here indicates longstanding functioning and patency of the lacrimal system through surgical intervention.

In conclusion, congenital punctal and canalicular atresia combined with congenital fistula is a treatable condition, using Teflon tubing and closure of the fistulous opening.
